# Fusion of electroencephalographic dynamics and musical contents for estimating emotional responses in music listening

**DOI:** 10.3389/fnins.2014.00094

**Published:** 2014-05-01

**Authors:** Yuan-Pin Lin, Yi-Hsuan Yang, Tzyy-Ping Jung

**Affiliations:** ^1^Swartz Center for Computational Neuroscience, Institute for Neural Computation, University of CaliforniaSan Diego, La Jolla, CA, USA; ^2^Center for Advanced Neurological Engineering, Institute of Engineering in Medicine, University of CaliforniaSan Diego, La Jolla, CA, USA; ^3^Music and Audio Computing Lab, Research Center for IT InnovationAcademia Sinica, Taipei, Taiwan

**Keywords:** EEG, emotion classification, affective brain-computer interface, music signal processing, music listening

## Abstract

Electroencephalography (EEG)-based emotion classification during music listening has gained increasing attention nowadays due to its promise of potential applications such as musical affective brain-computer interface (ABCI), neuromarketing, music therapy, and implicit multimedia tagging and triggering. However, music is an ecologically valid and complex stimulus that conveys certain emotions to listeners through compositions of musical elements. Using solely EEG signals to distinguish emotions remained challenging. This study aimed to assess the applicability of a multimodal approach by leveraging the EEG dynamics and acoustic characteristics of musical contents for the classification of emotional valence and arousal. To this end, this study adopted machine-learning methods to systematically elucidate the roles of the EEG and music modalities in the emotion modeling. The empirical results suggested that when whole-head EEG signals were available, the inclusion of musical contents did not improve the classification performance. The obtained performance of 74~76% using solely EEG modality was statistically comparable to that using the multimodality approach. However, if EEG dynamics were only available from a small set of electrodes (likely the case in real-life applications), the music modality would play a complementary role and augment the EEG results from around 61–67% in valence classification and from around 58–67% in arousal classification. The musical timber appeared to replace less-discriminative EEG features and led to improvements in both valence and arousal classification, whereas musical loudness was contributed specifically to the arousal classification. The present study not only provided principles for constructing an EEG-based multimodal approach, but also revealed the fundamental insights into the interplay of the brain activity and musical contents in emotion modeling.

## Introduction

Through monitoring ongoing electrical brain activity, electroencephalography (EEG)-based brain-computer interfaces (BCIs) allow users to voluntarily translate their intentions into commands to communicate with or control external devices and environments, instead of using conventional communication channels, e.g., speech and muscles (Millan et al., 2010). Several types of EEG signatures are theoretically defined and empirically proved to be robust in actively and reactively actuating BCIs (Zander and Kothe, 2011), such as evoked potentials, event-related potential (ERP), and sensorimotor rhythms (Wolpaw et al., 2002). Nowadays, a new categorization called passive BCI was introduced (Zander and Kothe, 2011). It enables users to involuntarily interact with machines by means of implicit user states, e.g., emotion. Researches are attempting to augment BCI's ability with emotional awareness and intelligence in response to users' emotional states, so called affective brain-computer interfaces (ABCIs).

Emotion is a psycho-physiological process as well as a natural communication channel of human beings. Music is considered as an extraordinary mediator to evoke emotions and concurrently modulate underlying neurophysiological processes (Blood et al., 1999). Upon profound findings in musical emotions, using machine-learning methods to characterize spatio-spectral EEG dynamics associated with emotions has gained increasing attentions in the last decade, namely EEG-based emotion classification, due to its promise of potential applications such as musical ABCI (Makeig et al., 2011), neuromarketing (Lee et al., 2007), music therapy (Thaut et al., 2009), implicit multimedia tagging (Soleymani et al., 2012a; Koelstra and Patras, 2013) and triggering (Wu et al., 2008). Given diverse EEG patterns, the major efforts in the previous EEG-based emotion classification works (not limited to music stimuli) were to seek an optimal emotion-aware model by leveraging feature extraction, selection and classification methods (Ishino and Hagiwara, 2003; Takahashi, 2004; Chanel et al., 2009; Frantzidis et al., 2010; Lin et al., 2010b; Petrantonakis and Hadjileontiadis, 2010; Koelstra et al., 2012; Soleymani et al., 2012b). Despite many approaches and advances in EEG analysis in the past decade, how to precisely categorize EEG signals into distinct emotional states remains challenging.

Music is an ecologically valid and complex stimulus that conveys emotions to listeners through compositions of musical elements, such as mode, tempo and timber (Peretz et al., 1998; Schmithorst, 2005; Gomez and Danuser, 2007; Zatorre et al., 2007). Listeners would be able to more and less perceive and recognize the same emotions as the music expresses (Schmidt and Trainor, 2001; Juslin and Laukka, 2003). Analogous to the EEG domain, researchers in music signal processing field devoted to map acoustic characteristics of musical contents into emotion semantics labeled by human annotators, namely music emotion recognition (Yang and Chen, 2011, 2012). Most of previous works employed publicly available toolboxes, such as MIRToolbox (Lartillot and Toiviainen, 2007), Marsyas (Tzanetakis and Cook, 2002), and PsySound (Cabrera, 1999), to extract a wide variety of musical features and then used machine-learning algorithms to automatically learn the associations between extracted features and emotions expressed in music (Yang et al., 2008a; Aljanaki et al., 2013). The aforementioned evidence raises a natural question whether or not the acoustic characteristics of musical contents can further improve the EEG classification results.

Using EEG features in conjunction with other information sources recently shed light on this issue, for example peripheral biosignals (Chanel et al., 2009; Koelstra et al., 2012; Soleymani et al., 2012a), eye gaze (Soleymani et al., 2012a,b), musical structures (Koelstra et al., 2012), and facial expression (Koelstra and Patras, 2013) have been proposed. Particularly for the music study, Koelstra et al. (2012) reported that a multimodal approach, fusing decision outputs from EEG and music classifiers, marginally improved the classification performance over using solely EEG modality. It remained unclear whether or not the acoustic characteristics of musical contents effectively contribute to the emotion modeling.

This study attempted to examine the roles of EEG and music modalities in the multidiscipline emotion classification problem in music listening upon two posed hypotheses. The first hypothesis was that the EEG modality reflecting spatio-spectral brain activities of the whole brain about implicit emotion responses should dominate the multimodal approach for emotion classification, as compared to the music modality, in which the implicit emotions concerned the responses automatically induced by the stimulus itself (Gyurak et al., 2011). This study adopted machine-learning methods, i.e., feature extraction, selection, and classification, to systematically assess a composite feature space synchronizing EEG dynamics and musical characteristics in accordance to time scale. The relative contributions from EEG and music modalities then can be explored. Furthermore, one can imagine that the use of a high-density EEG montage over the whole head might be more difficult or impractical for real-life ABCI applications. The applicability of whole-head EEG dynamics (in the first hypothesis) might no longer hold if only few electrodes are available over a certain region or regions (Lin et al., 2010b). Thus, this study posed another hypothesis that the musical contents might complement less informative EEG dynamics for emotion classification and consequently improve over the EEG modality result. This study explored the minimal set of informative electrodes from multiple subjects for emotion classification. Such few electrodes mostly populated over the fronto-central regions were used to simulate the absence of whole-brain EEG dynamics. Exploring the validity of these two hypotheses might elucidate potential advantages and limitations in fusing EEG dynamics and musical contents for the emotion classification problem.

## Materials and methods

### EEG dataset and music excerpts

**Figure 1:**
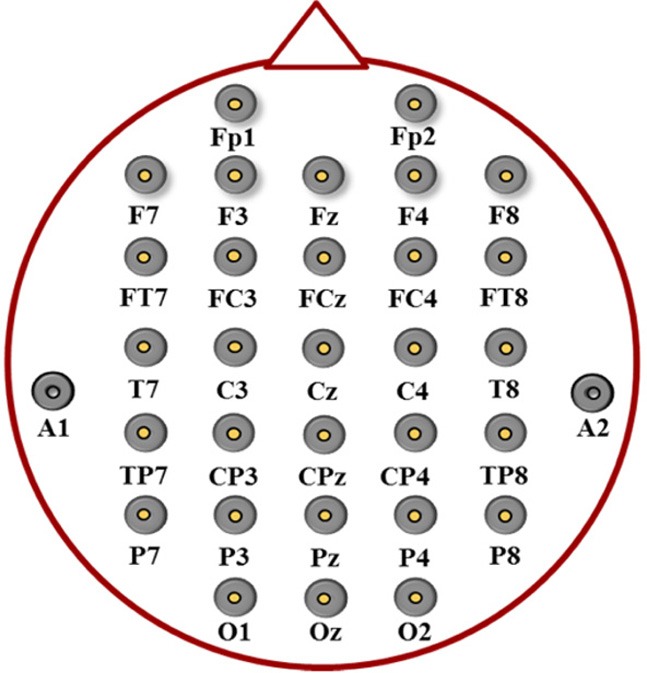
**Electrode placements of 32 channels according to the international 10–20 system**.

This study adopted the Oscar movie soundtrack dataset (Lin et al., 2010b) to test the feasibility of using a multimodal approach for emotion classification. The EEG signals were collected from 26 healthy subjects who were undergraduate and graduate students (16 males, 10 females; age 24.40 ± 2.53) mostly from engineering-related colleges. The experiment protocol and EEG recording were approved by the Human Research Protections Program of National Taiwan University. The music-listening experiment targeted four emotion classes (joy, anger, sadness, and pleasure) in accordance to the two-dimensional circumplex emotion model composed of valence (positive-negative) and arousal (high-low) axes (Russell, 1980). Sixteen music excerpts from the soundtracks of Oscar winning movies were used to induce the targeted emotions. Each subject underwent a 4-block music experiment; each block contained four counterbalanced 30-s music trials corresponding to four targeted emotions. After music listening, the subjects labeled their felt emotions on a discrete scale, for example, joy (positive valence and high arousal), anger (negative valence and high arousal), sadness (negative valence and low arousal), and pleasure (positive valence and low arousal). In the experiment, a 32-channel Neuroscan EEG module placed according to the International 10–20 system (Figure 1) and referenced to the linked mastoids (algebraic average of left and right) was adopted to acquire EEG signals with a sampling rate of 500 Hz and a bandpass filter at 1–100 Hz. Subjects were asked to keep their eyes closed, remain seated, and minimize head/body movements. After the music experiment, each subject's data was consisted of 16 30-s EEG segments labeled by self-reported emotional states (joy, anger, sadness, or pleasure).

Referring to the recent works (Koelstra et al., 2012; Soleymani et al., 2012a,b; Koelstra and Patras, 2013), most of EEG-based classification tasks addressed and performed on the basis of emotional valence and arousal, e.g., categorizing EEG signals into positive or negative valence, instead of discrete emotion states. To make a direct comparison with the latest reports, this study addressed the binary emotion classification problem. The self-reported emotion labels of the Oscar movie soundtrack dataset were separately merged into the binary categories of valence and arousal. The valence scale comprised positive (joy and pleasure) and negative (anger and sadness) levels, whereas arousal scale contained high (joy and anger) and low (pleasure and sadness) levels. There were 16 pairs of 30-s EEG signals and music excerpts for each of 26 subjects available for analysis and comparison.

### EEG feature extraction

Previous neurophysiological studies documented EEG spectral changes either in distinct regions or between hemispheres (Davidson, 1992; Schmidt and Trainor, 2001; Aftanas et al., 2004; Sarlo et al., 2005; Sammler et al., 2007). Such evidence might in part facilitated the use of spectral dynamics within/between channels for the EEG-based emotion classification, e.g., spectra in individual channels and spectral asymmetry in left-right channel pairs (Lin et al., 2010b; Koelstra et al., 2012; Soleymani et al., 2012a; Koelstra and Patras, 2013). In the literature, the patterns of spectral differences along anterior and posterior brain regions have also been explored (Schmidt and Trainor, 2001; Sarlo et al., 2005). However, no study has attempted to address the feasibility of using spectral differences in fronto-posterior channel pairs in this domain. Prior to construct a multimodality approach, this study aimed to explore an optimal EEG features from different types, including the power spectral density in individual channels and the power spectral asymmetry in the left-right and fronto-posterior channels pairs.

For each of 16 30-s EEG trials, the short-time Fourier transform with non-overlapping 1-s Hamming window was applied to extract the power spectral density in five frequency bands, including delta (δ: 1–3 Hz), theta (θ: 4–7 Hz), alpha (α: 8–13 Hz), beta (β: 14–30 Hz), and gamma (γ: 31–50 Hz) over 30 channels (two reference channels were excluded). The band-specific power spectra of the individual channels formed a feature dimension of 150 (5 bands × 30 channels) and was labeled as PSD hereafter. To characterize the spectral-band asymmetry in respect of laterality (in left-right direction) and caudality (in fronto-posterior direction), this study defined two feature types namely DLAT and DCAU to separately extract the differential spectral asymmetry of 12 left-right and 12 fronto-posterior channel pairs from 30 individual channels, both forming a feature dimension of 60 (5 bands × 12 channel pairs). Furthermore, this study also named a feature type MESH by merging PSD, DLAT and DCAU, a dimension of 270, for comparison. Table 1 summarizes the aforementioned four EEG feature types. It is noted that the feature vectors of each type were separately normalized to the range from 0 to 1.

**Table 1:** **A summary of EEG feature types**.

**Type**	**# Electrodes**	**# Features**	**Extracted features**
DLAT	24	60	Five differential spectral band power (δ, θ, α, β, and γ) for 12 left-right electrode pairs: Fp1-Fp2, F7-F8, F3-F4, FT7-FT8, FC3-FC4, T7-T8, P7-P8, C3-C4, TP7-TP8, CP3-CP4, P3-P4, and O1-O2.
DCAU	24	60	Five differential spectral band power (δ, θ, α, β, and γ) for 12 fronto-posterior electrode pairs: Fp1-O1, Fp2-O2, F7-P7, F3-P3, Fz-Pz, F4-P4, F8-P8, FT7-TP7, FC3-CP3, FCz-CPz, FC4-CP4, and FT8-TP8.
PSD	30	150	Five spectral band power (δ, θ, α, β, and γ) for 30 electrodes: Fp1, Fp2, F7, F3, Fz, F4, F8, FT7, FC3, FCz, FC4, FT8, T7, C3, Cz, C4, T8, TP7, CP3, CPz, CP4, TP8, P7, P3, Pz, P4, P8, O1, Oz, and O2.
MESH	30	270	A combination of DLAT, DCAU, and PSD.

### Music feature extraction

Emotion expression in music is usually associated with different acoustic characteristics (Juslin, 2000; Gabrielsson and Lindström, 2010). This study employed commonly used music information retrieval toolboxes, i.e., MIRtoolbox (Lartillot and Toiviainen, 2007) and PsySound (Cabrera, 1999), to extract the acoustic features that represent various perceptual dimensions of music listening, including pitch, dissonance, loudness, and timber. The data samples of the musical features were aligned to the EEG features with one sample per second. The music feature types are summarized in Table 2 and depicted as followings.

**Table 2:** **A summary of music feature types**.

**Type**	**# Features**	**Extracted features**
Pitch	3	Key clarity, Mode, Harmonic flux
Dissonance	4	Tonal dissonance (HK,S), Spectral dissonance (HK, S)
Loudness	5	Loudness, Sharpness (Z, A), Timbral width, Volume
MFCC	13	MFCC coefficients (13 features)
MUSIC	25	A combination of Pitch, Dissonance, Loudness, and MFCC

Pitch is the auditory attribute of sounds which can be ordered on a scale from low to high. The harmonic aspect of music can be described in terms of the relationship between two or more simultaneous pitches, whereas the melodic aspect is related to the temporal succession of pitches (Muller et al., 2011). This study used the MIRtoolbox to extract three major elements describing the pitch properties in music, including the key clarity, mode, and harmonic flux. The key clarity refers to the similarity (or key strength) that best describes one of the 24 musical keys, e.g., C major. Next, the musical mode represents the difference between the best major key and the best minor key in key strength, which is often related to the sensation of valence in music (Gabrielsson and Lindström, 2010). The harmonic flux indicates a large difference in harmonic content between consecutive frames, such as chord changes, strong melody or bass line movement. This feature may be relevant as some psychology studies have found that large melodic intervals are perceived as more powerful (i.e., high-arousal) than small ones (Gabrielsson and Lindström, 2010).

Dissonance measures the harshness or roughness of the acoustic spectrum (Cabrera, 1999). The dissonance generally implies a combination of notes that sound harsh or unpleasant to people when played at the same time. Empirically, many musical pieces involve a balanced combination of consonance and dissonance sounds, e.g., the release of harmonic tension might create pleasure (Parncutt and Hair, 2011). Four elements describing the dissonance were calculated by the PsySound, including tonal dissonance (HK and S) and spectral dissonance (HK, S). The tonal and spectral dissonance measures the dissonance among tonal components and models the degree deviating from the noisiness of the sound, respectively. Note that HK and S are two methods forming the results in different scales.

Loudness is the perceptual intensity of sounds and depends primarily on the physical intensity as well as frequency and duration. This study employed the PsySound to derive five features depicting the human sensation of sound loudness across frequency, including loudness, sharpness (Z, A), timbral width, and volume. The loudness is an integral of the spectral distribution of loudness sensation. In general, loud music tends to be associated with high arousal and potency, whereas soft music relates to low arousal. Next, sharpness Z and A are two models distinctly characterizing the sharpness of the sound sensation in a scale from dull to sharp (Cabrera, 1999). The former model emphasizes high frequencies, whereas the later one is sensitive to the positive influence of loudness toward sharpness. The timbral width is defined as the flatness, i.e., width of the peak, of the loudness' spectral distribution, whereas the volume is derived based on the relative strength between total loudness and sharpness (Cabrera, 1999). The relationship between these two features and emotion processing is relatively less understood.

Timber that reflects the acoustic spectro-temporal characteristics is often considered as the quality of sound that makes a particular musical sound different from another. To model the timber, this study employed the MIRToolbox and computed the Mel-frequency cepstral coefficients (MFCC). MFCC characterizes the spectral shape of the sound by taking the coefficients of the discrete cosine transform of log-power spectra expressed on a non-linear perceptual-related Mel-frequency scale (Davis and Mermelstein, 1980). Typically, only the 10–20 lowest coefficients were retained for analysis (Muller et al., 2011). Referring to (Koelstra et al., 2012), this study only adopted the first 13 coefficients. The timber type was named as MFCC hereafter.

### Fusion of EEG and musical features

Through using multidisciplinary signals, a multimodal approach can usually boost single modality results. Decision-level and feature-level fusions are two commonly used schemes to obtain the integration of multiple signal sources (Kittler et al., 1998; Sargin et al., 2007). The feature-level fusion works by concatenating features of different modalities and then feeding the composite feature vector to a classifier, whereas the decision-level fusion allows single modalities to process independently and then derive a final decision from multiple outputs. It is worth noting that since this study attempted to evaluate the relative contributions of EEG and music modalities, the feature-level fusion that synchronizes the features of different modalities along time more likely conforms to the objective. After applying a feature selection processing (described at the next section), this study defined a term, namely percent composition, to reveal the percentages of contributions of EEG and musical features to a multimodal feature composition. Prior to classification, each of the addressed EEG and musical features was independently normalized between 0 and 1, making features equally weighted to a classifier.

### Feature selection

Feature selection plays a chief role in solving classification problem. Given a plenty of raw features, the selection procedure is capable of extracting only a subset of task-relevant features while removing redundant/irrelevant ones. Feature reduction not only leads to computational efficiency, but also reduces the number of electrodes required in real-life applications (Lin et al., 2010b). This study employed an F-score index, a ratio of between- and within-class variations (Chen and Lin, 2006), to pinpoint the most emotion-relevant features/electrodes, which has been proven effective for the EEG-based emotion classification problem (Lin et al., 2010b). The F-score index of the *i*th feature is defined as following:

$$F(i)=\frac{\sum_{l=1}^{g}{\left({\bar{x}}_{l,i}-{\bar{x}}_{i}\right)}^{2}}{\frac{1}{n_{l}}\sum_{l=1}^{g}\sum_{k=1}^{n_{l}}{\left({\bar{x}}_{k,l,i}-{\bar{x}}_{l,i}\right)}^{2}}$$

where *x_i_* and *x_l,i_* are the mean values of the *i*th feature for entire dataset and for class *l* (*l* = 1 ~ *g*, *g* = 2 for positive and negative classes in valence or high and low classes in arousal), respectively; *x_k,l,i_* is the *k*th sample value of the *i*th feature for class *l*, and *n_l_* is the number of samples in class *l*. The larger F-score value indicates higher discrimination power. It assumed that the features with highest F-score values account for the most emotion-tagged information and contribute more to emotion classification.

To test the first hypothesis, the F-score based feature selection was applied to each subject's EEG dataset separately to generate a subject-dependent EEG feature set. To test the second hypothesis, this study simulated the consequences of unavailability of whole-head EEG data. This study applied the F-score feature selection to explore the commonality of the informative EEG features from 26 subjects, i.e., subject-independent set. More specifically, an objective index, namely the level of feature independency (LFI), was defined as the number of subjects having the same informative features. After sorting and accumulating the F-score-sorted subject-dependent EEG features, the LFI-guided subject-independent EEG feature sets were then explored. The LFI value was empirically set and tested from 0.1 up to 0.6. Note that no informative features were commonly observed over 18 subjects (LFI = 0.7). The subject-independent EEG feature set with LFI = 0.6 was supposed to return a minimal set of electrodes to test the second hypothesis. It is also important to explore the common EEG patterns across subjects in emotion processing.

### Feature classification and validation

Support vector machine (SVM) is a popular machine-learning algorithm that projects input data onto a higher dimensional feature space via a transfer kernel function, in which classification can be made more easily than in the original feature space. The iterative learning processing of an SVM eventually converges into optimal hyperplanes giving maximal margins between classes. This study used LIBSVM software (Chang and Lin, 2011) to build the SVM classifier and employed a radial basis function (RBF) kernel to non-linearly map the original data onto a higher dimensional space.

Regarding the classification validation, this study adopted a leave-trial-out (LTO) validation method to each individual's dataset to obtain the emotion classification results. The LTO validation provides a generalized performance by averaging classification results *N* times with each of *N* trials to be tested (*N* = 16 in this study). In each repetition, the SVM model was trained with 15 trials and then tested against the remaining trial. It is noted that prior to the LTO validation a grid-search procedure (Chang and Lin, 2011) was applied to the entire dataset to decide an optimal parameter pair (γ, *C*) for the size of the RBF kernel and the penalty of decision boundary from various pairs (γ: 2^−1^~2^3^, *C*: 2^−4^~2^1^), which corresponded to the best SVM training accuracy. The classification accuracy was defined by the ratio of correctly classified number of samples and the total number of samples. The averaged classification performance was obtained by averaging the classification results across 26 subjects. This study employed a paired *t*-test to access the statistical significance in classification performance between different feature types or modalities. As a baseline, the majority-voting accuracy defined by the majority class of the training data was also provided, i.e., random guessing. For example, given a training set consisted of positive (63%) and negative (37%) samples in the valence classification, the majority accuracy was 63% for assigning a new sample as positive valence. The significant difference of the obtained classification accuracy versus majority voting was tested using a one-sample *t*-test.

## Results

### Testing the first hypothesis: EEG dynamics dominated a multimodal approach in emotion classification compared to musical contents

**Figure 2:**
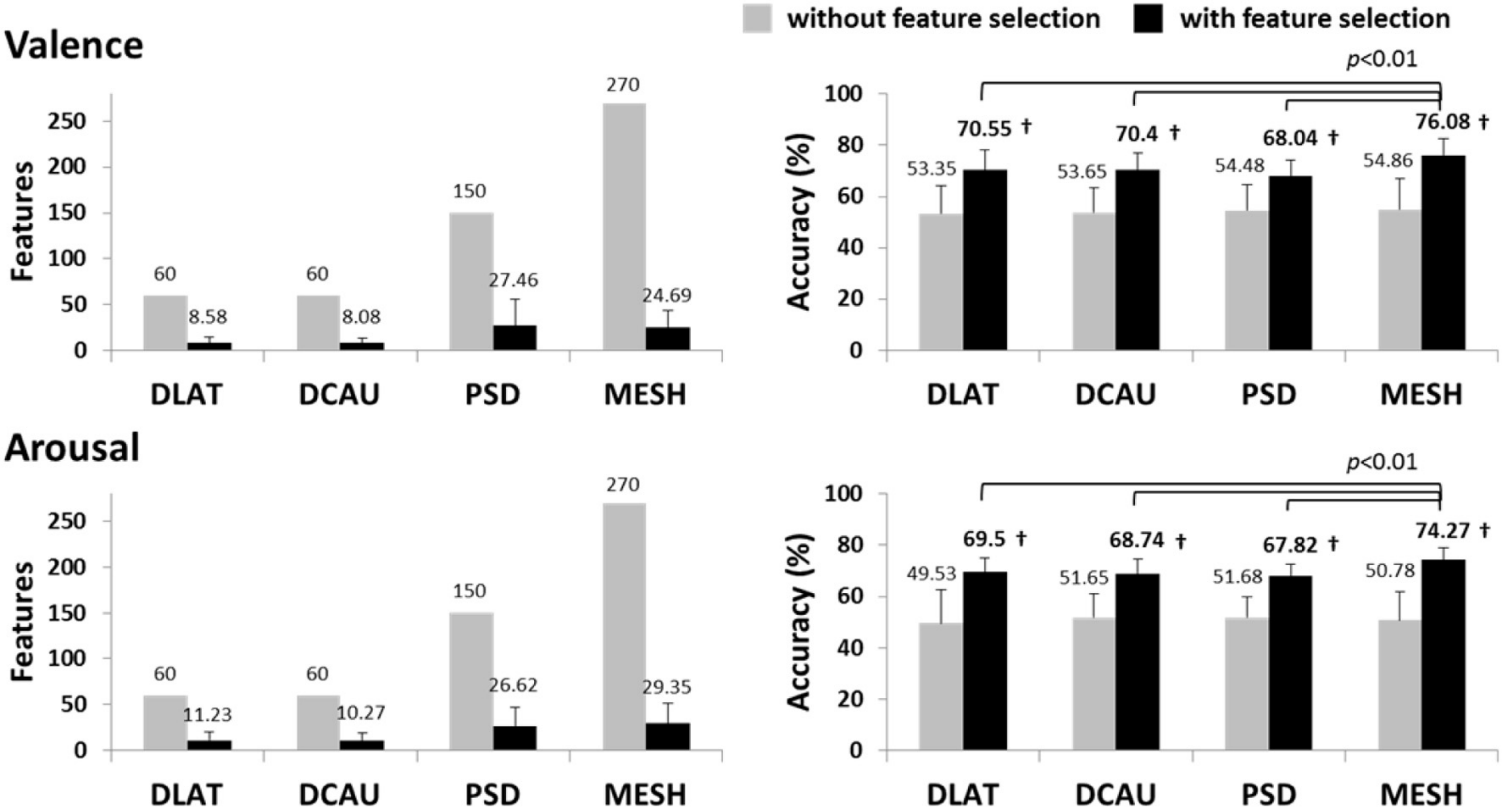
**The valence and arousal classification results using the subject-dependent EEG feature sets with/without the F-score based feature selection**. The numbers above the bars represent the mean values of the results, whereas the numbers in bold indicate the accuracies significantly better (*p* < 0.01) than the majority voting accuracy (valence: ~63%, arousal: ~61%). ^†^Indicates that the accuracy with feature selection significantly outperformed that without feature selection (*p* < 0.01).

Figure 2 summarizes the valence and arousal classification results of the subject-dependent EEG feature types (DLAT, DCAU, PSD, and MESH). It is noted that the condition “without feature selection” shows the results using all the features, while the condition “with feature selection” shows the maximum accuracy through the add-one-feature-in procedure and the number of the features eventually used. In general, using different EEG feature types without the feature selection tended to have comparable results that were notably worse than majority voting. Using only informative features (with high F-score values), the classification accuracies for all the feature types were markedly improved (*p* < 0.01) upon the results without using feature selection, and were significantly better than the majority voting (*p* < 0.01). The MESH generated maximum accuracies of 76.08 ± 6.39% and 74.27 ± 4.82% for valence and arousal classification, respectively, which significantly outperformed other feature types (*p* < 0.01). The feature selection also considerably reduced the feature dimensionality from 270 to below 30. This was very likely attributed to the fact that the F-score feature selection effectively pruned the less informative features from the whole feature space, largely alleviating the interference caused by redundant/irrelevant features. Thus, the MESH was used to merge with musical contents to form a multimodal approach in the following sections.

**Figure 3:**
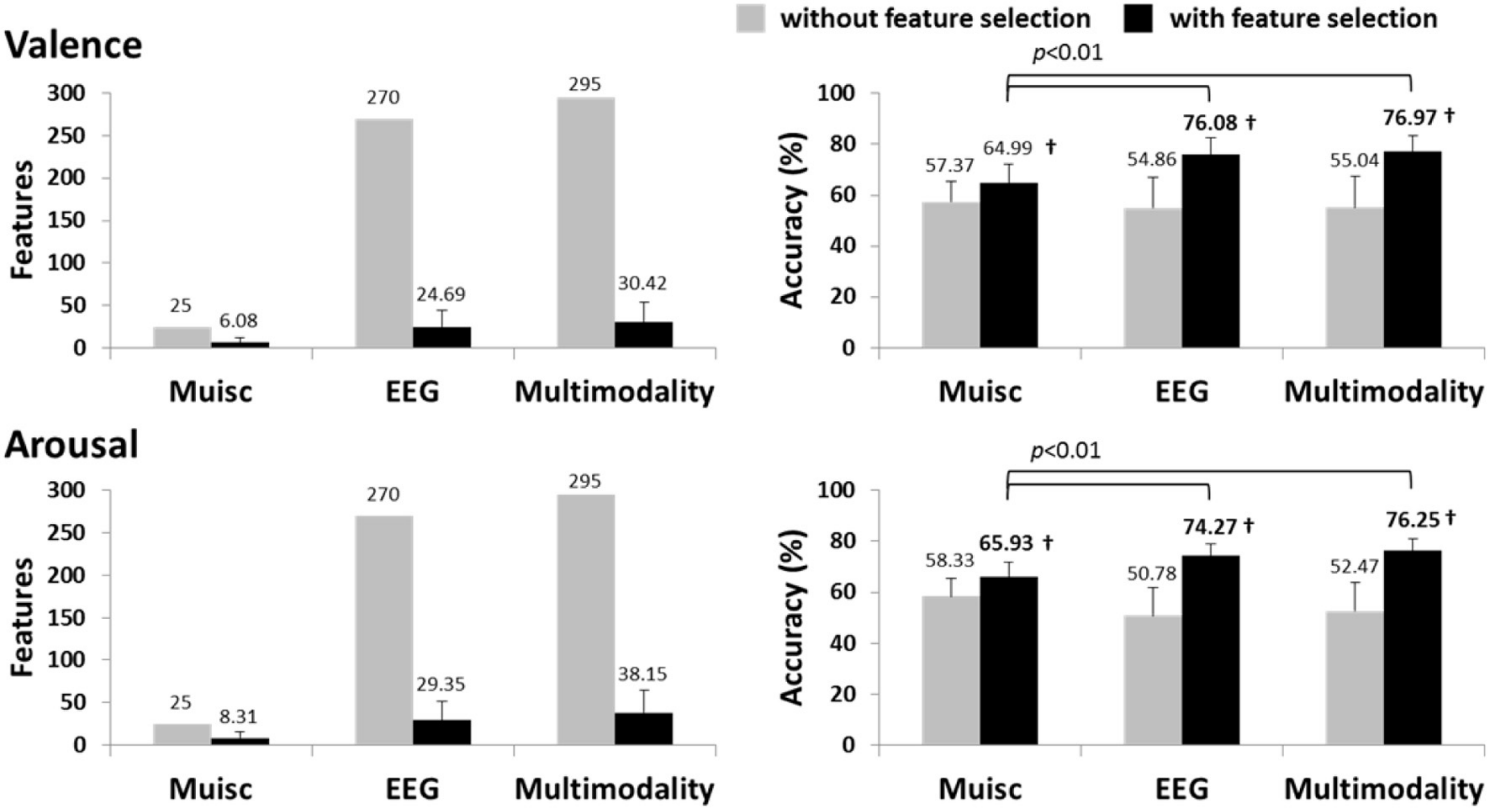
**The valence and arousal classification results using the subject-dependent multimodal approach with/without feature selection**. The results of the subject-dependent EEG modality (feature type: MESH) and the music modality (feature type: MUSIC) are also provided for comparison. The numbers above the bars represent the mean values of the results, whereas the numbers in bold indicate the accuracies significantly better (*p* < 0.01) than the majority voting accuracy (valence: ~63%, arousal: ~61%). ^†^Indicates that the accuracy with feature selection significantly outperformed that without feature selection (*p* < 0.01).

Figure 3 summarizes the classification results using the subject-dependent EEG features (i.e., MESH), musical features (i.e., MUSIC), and subject-dependent multimodal approach. Note that the multimodal features were obtained by applying the F-score feature selection to the composite features of the MESH and MUSIC features. The multimodal approach obtained the maximum accuracies of 76.97 ± 6.18% and 76.25 ± 4.88% for valence and arousal classification, respectively. The results using musical features alone were around 65% and only significantly outperformed the majority voting for arousal classification but not for valence classification, which were all significantly worse than EEG and multimodality approaches (*p* < 0.01). The subject-dependent EEG features did not notably benefit from the inclusion of musical features. The classification performance using the multimodal features compared favorably (*p*>0.1) to those using the EEG features.

**Figure 4:**
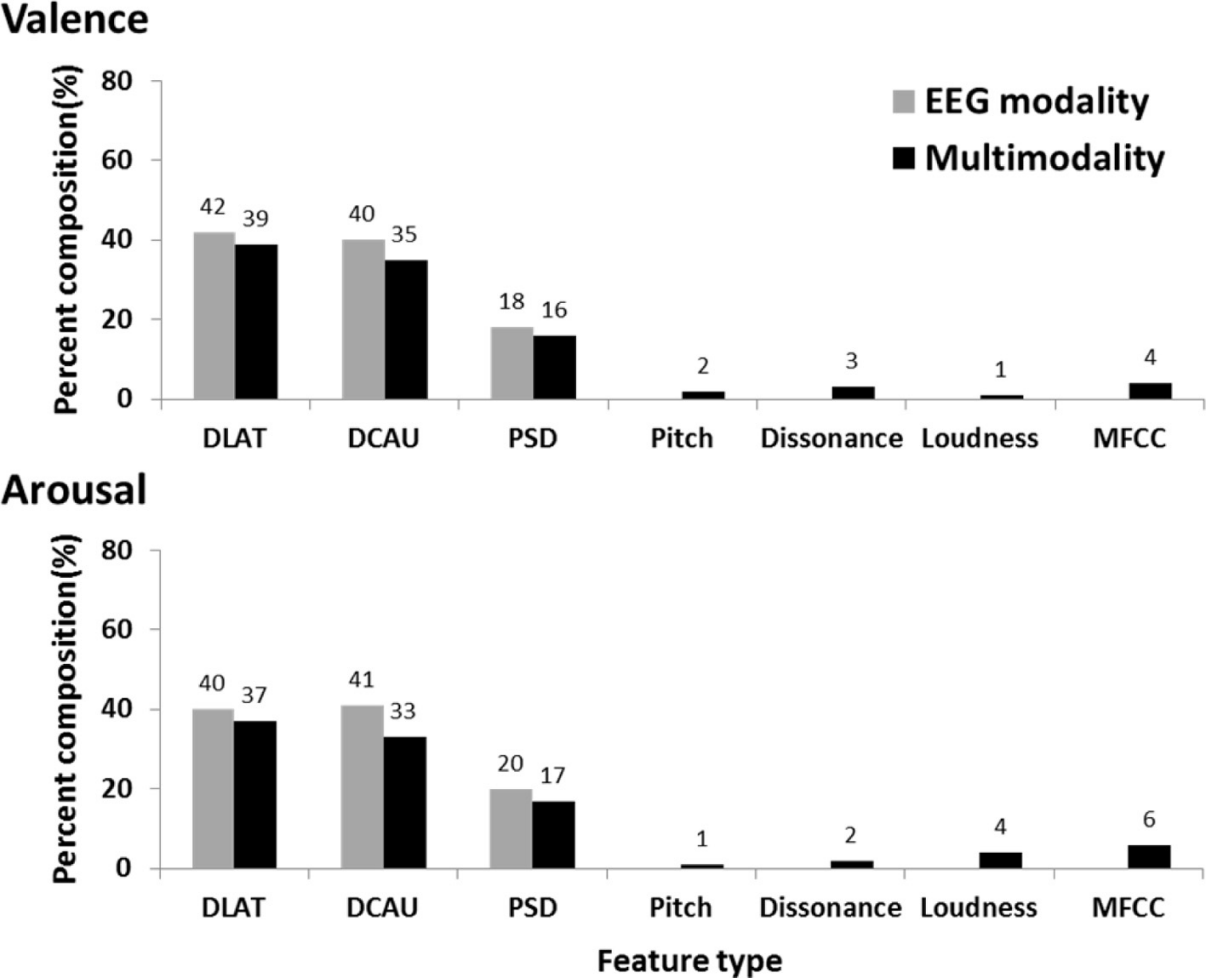
**The percent composition of contributions of EEG (DLAT, DCAU, and PSD) and musical (Pitch, Dissonance, Loudness, and MFCC) features to the subject-dependent multimodality**. The composition of the subject-dependent EEG modality is also provided for comparison.

Figure 4 further shows the percent composition of contributions of EEG and musical features to the subject-dependent multimodal approach. The composition was derived based on how many informative features led to the maximum classification accuracy. This result indicated that the EEG feature types, especially DLAT and DCAU, dominated the composition of multimodal features for valence and arousal classifications, while the musical features barely contributed. This might explain the marginal improvement using the multimodal approach versus the EEG-only modality.

In sum, the feature type MESH, consisted of the two-directional power asymmetry and individual power spectra across the whole scalp and frequency bands, better characterizing the EEG dynamics about emotional responses than the musical features. The above empirical results proved the first hypothesis that the EEG modality that accessed spatio-spectral brain activity of the whole brain dominated the classification of emotional responses in the multimodal approach.

### Testing the second hypothesis: musical contents can complement EEG dynamics unavailable in whole-head EEG recordings

To test the second hypothesis that musical contents can complement EEG dynamics unavailable in whole-head EEG recordings, this study simulated the circumstance of classifying emotion states based on fewer informative EEG features/electrodes. The LFI index (0.1~0.6) was defined to systematically reduce the whole-brain electrode montage (30) to different subsets of electrodes located at certain regions. Under such constrain, the relationship between the EEG dynamics and musical contents can be evaluated.

**Figure 5:**
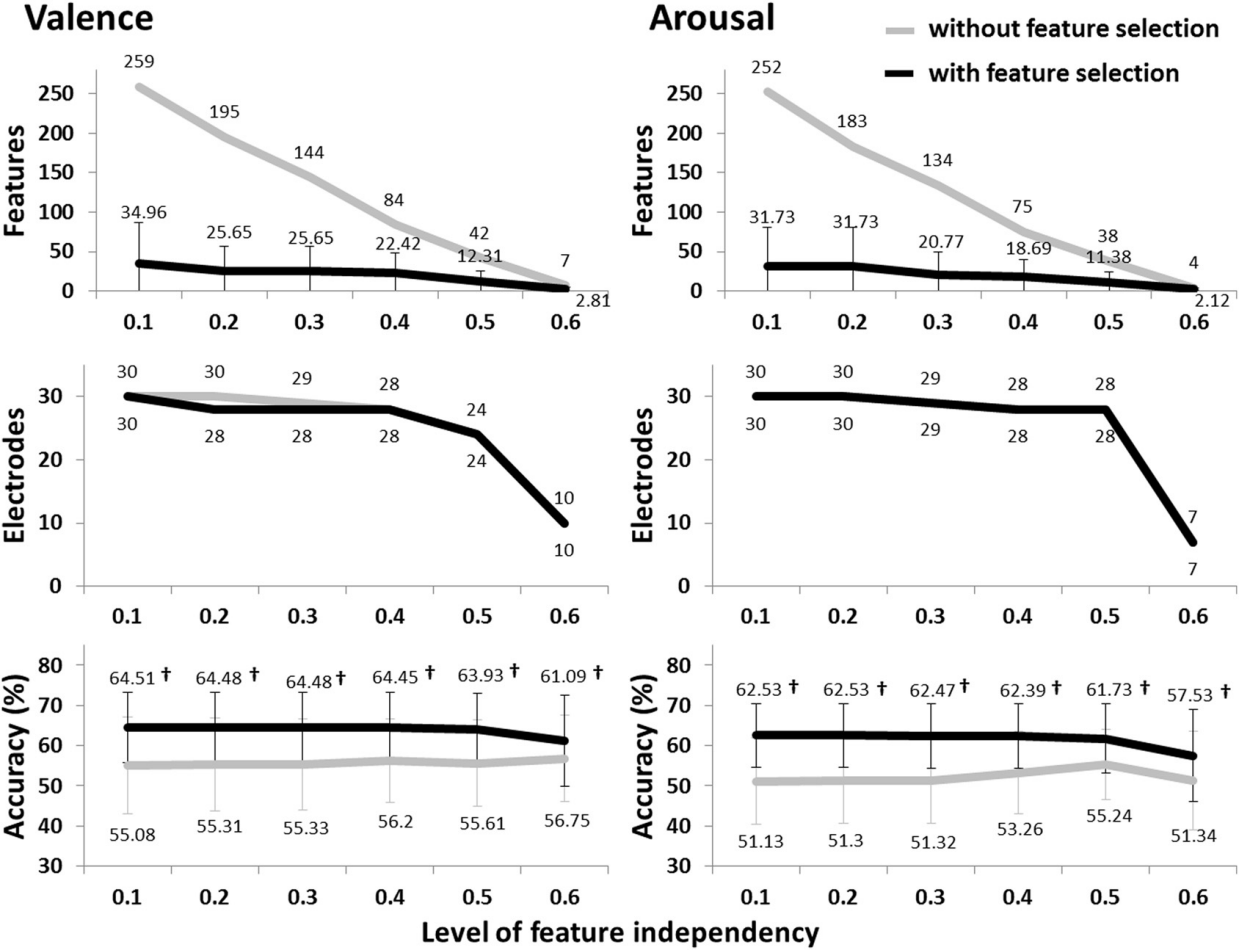
**The valence and arousal classification results of the subject-independent EEG features (type: MESH) in term of the average number of features, electrodes, and accuracies using with/without feature selection under the LFI criteria (0.1 ~ 0.6)**. The numbers near to the nodes represent the mean values of the results. ^†^Indicates that the accuracy with feature selection significantly outperformed that without feature selection (*p* < 0.01), yet were comparable (*p* > 0.1) to majority voting accuracies (valence: ~63%, arousal: ~61%).

**Figure 6:**
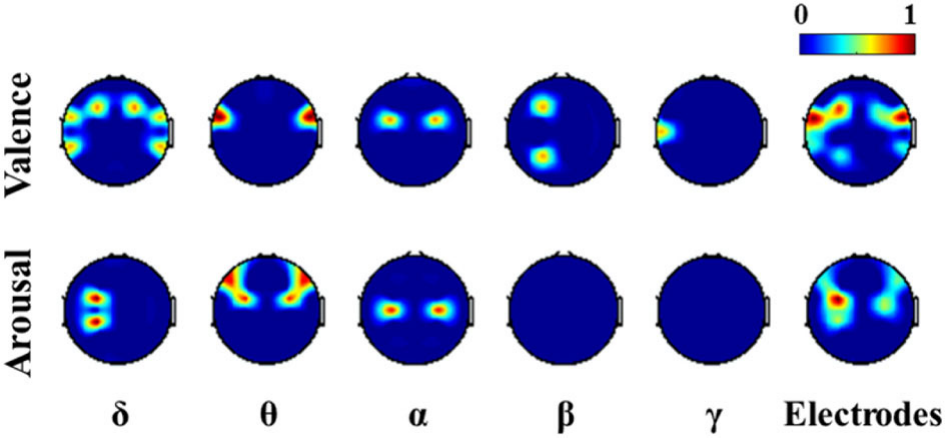
**The topographic mapping of informative EEG features consistently appeared in multiple subjects**. The rightmost topography color-codes the importance of electrodes according to how frequent the electrodes were used to derive the corresponding features.

Figure 5 presents the valence and arousal classification results using the LFI-sorted subject-independent EEG features (i.e., MESH) with/without feature selection. Overall, the number of features can be seen to progressively reduce as the LFI value increased from 0.1 to 0.6. The number of electrodes required for the feature sets in turn was reduced. These feature sets, however, gave very limited estimations in emotional responses against the majority voting. The reason was attributed to the fact that the discriminative power of the subject-independent features with a compromise of a subject population might not be guaranteed to each of subjects. At LFI = 0.6, the required electrodes were dramatically reduced from the whole-scalp montage (30) to ten and seven electrodes for valence and arousal classification, respectively. As shown in Figure 6, most of the informative EEG features (listed in Table 3) were extracted from the fronto-central electrodes versus others. It is worth noting that the DLAT, extracted from left-right electrode pairs, dominated the composition of the EEG features, compared to others (DCAU and PSD). According to these results, the subject-independent EEG feature set (LFI = 0.6), which involved a low-density fronto-central montage, was adopted for emotion classification in the rest of the study.

**Table 3:** **The informative EEG features that consistently appeared across multiple subjects**.

**Rank**	**Valence**	**Arousal**
1	DLAT: FT7-FT8 (Theta)	DCAU: FC3-CP3 (Delta)
2	DLAT: FC3-FC4 (Alpha)	DLAT: C3-C4 (Alpha)
3	DLAT: F3-F4 (Delta)	DLAT: F7-F8 (Theta)
4	DLAT: FT7-FT8 (Delta)	DLAT: FC3-FC4 (Theta)
5	DLAT: TP7-TP8 (Delta)	
6	DCAU: F3-P3 (Beta)	
7	PSD: T7 (Gamma)	

**Figure 7:**
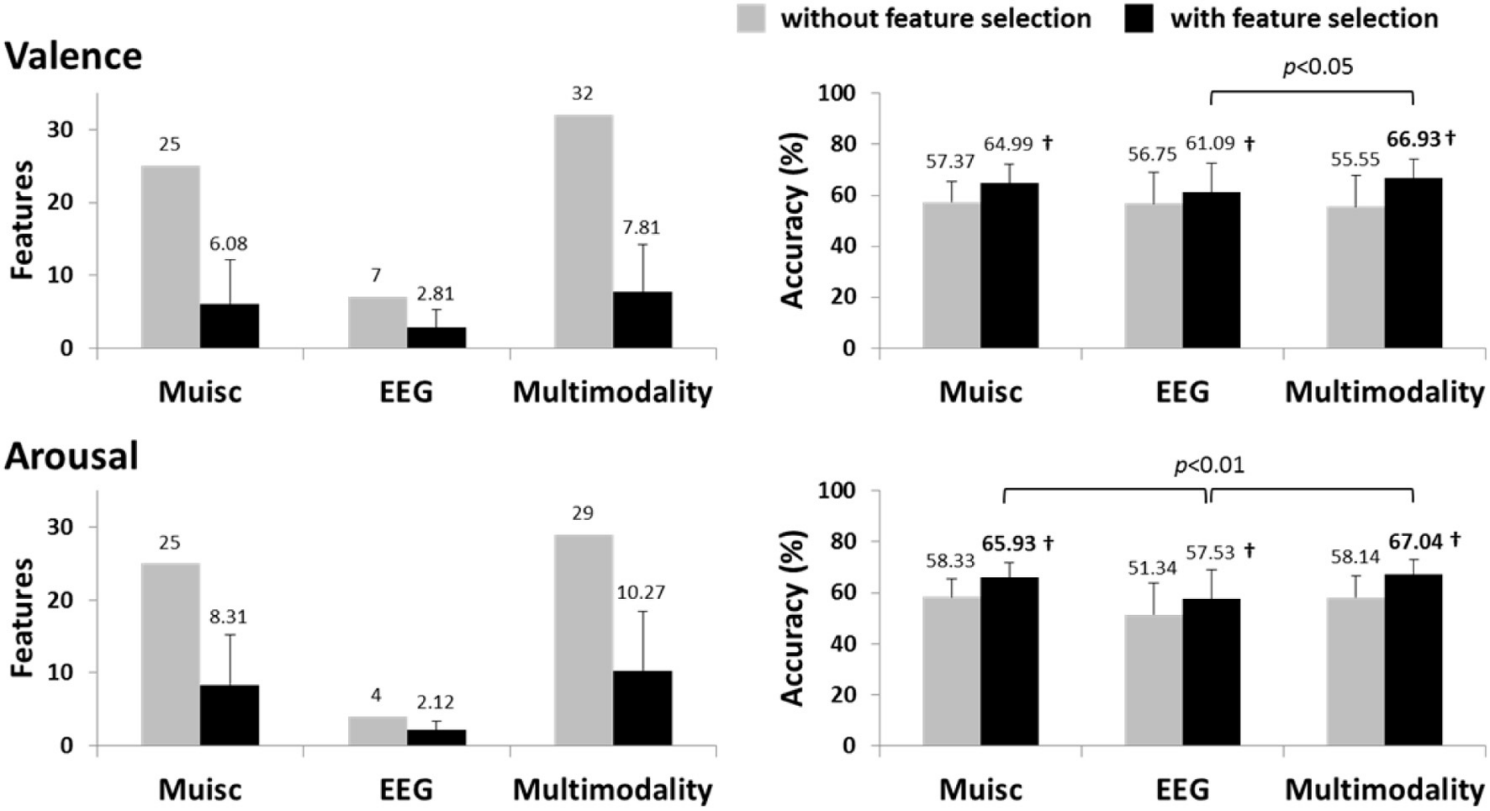
**The valence and arousal classification results using the subject-independent multimodal approach (LFI = 0.6) with/without feature selection**. The results of the subject-independent EEG modality (feature type: MESH) and the music modality (feature type: MUSIC) are also provided for comparison. The numbers above the bars represent the mean values of the results, whereas the numbers in bold indicate the accuracies significantly better (*p* < 0.02) than the majority voting accuracy (valence: ~63%, arousal: ~61%). ^†^indicates that the accuracy with feature selection significantly outperformed that without feature selection (*p* < 0.01).

Figure 7 shows the classification results using the subject-independent EEG features (i.e., MESH given LFI = 0.6), musical features (i.e., MUSIC), and subject-independent multimodal approach. Note that the sorted multimodal features were derived by applying the F-score feature selection to the composite feature vector of the MESH and MUSIC features. The multimodal approach resulted in the maximum accuracies of 66.93 ± 7.10% and 67.04 ± 5.78% for valence and arousal classification, respectively, following by the musical features and the EEG features. Most importantly, the multimodal approach outperformed the EEG-only features by around 6% for valence (*p* < 0.05) and 9% for arousal (*p* < 0.01) classification. There was no significant difference between the multimodal approach and the musical features (*p* > 0.3).

**Figure 8:**
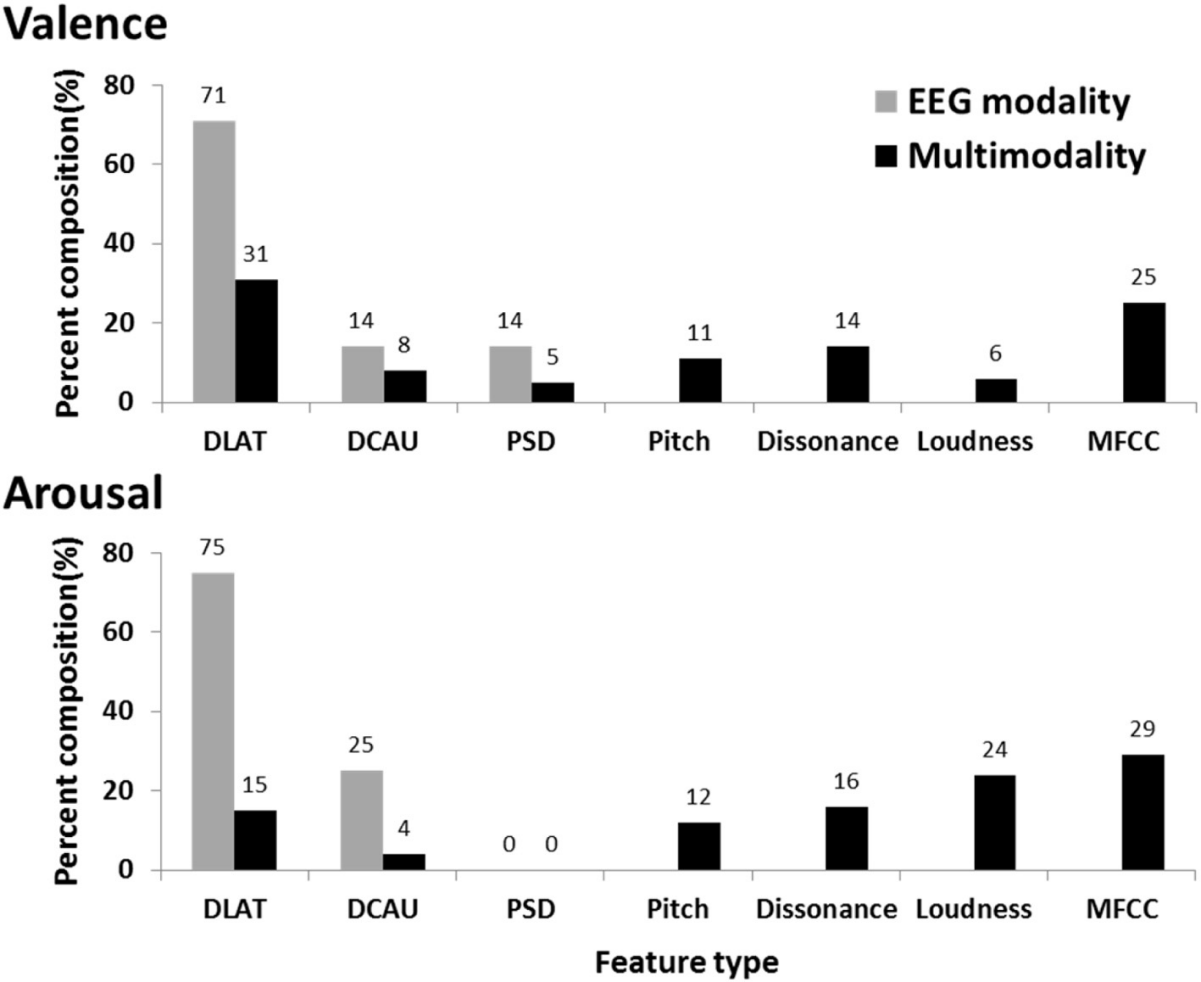
**The percent composition of contributions of EEG (DLAT, DCAU, and PSD) and musical (Pitch, Dissonance, Loudness, and MFCC) features to the subject-independent multimodality**. The composition of the subject-independent EEG modality is also provided for comparison.

Figure 8 shows the percent composition of contributions of EEG and musical features to the subject-independent multimodal features. As a baseline, the composition of the subject-independent EEG features is also provided. The comparative result showed the EEG and musical features performed complementarily in the multimodal approach. The musical features competed to the EEG features and replaced the ones with relatively low discriminative power, especially for arousal scale. This evidently explains the reason that the subject-independent multimodal approach leading to significant improvements upon the subject-independent EEG results. Table 4 lists these informative musical features, which consistently appeared in above half of the subjects.

**Table 4:** **The informative musical features in the subject-independent multimodal approach**.

**Rank**	**Valence**	**Arousal**
1	Dissonance: Spectral dissonance (S)	Loudness: Sharpness (Z)
2	Pitch: Mode	MFCC: 8th
3		Loudness: Sharpness (A)
4		Pitch: Harmonic flux

In sum, the EEG features extracted from a subset of brain regions was unable to effectively encompass the complex brain dynamics about emotions. The corresponding EEG features simply retuned the classification accuracy equivalent to random guessing. Under this circumstance, the EEG modality could benefit from the inclusion of the acoustic characteristics of musical contents. The aforementioned simulation result proved the second hypothesis that the musical contents can compensate for EEG dynamics unavailable in whole-head EEG recordings to improve the classification performance to some extent.

## Discussion

Music is an ecologically valid and complex stimulus that conveys emotions to listeners through musical composition. Using only EEG signals to classify music-induced emotional responses remained challenging. By exploiting the complementary nature of multidisciplinary modalities, the fusion of EEG and musical dynamics has been recently reported (Koelstra et al., 2012). However, it remains unclear when acoustic characteristics of musical contents effectively contribute to the modeling of emotional responses. To this end, this study adopted machine-learning methods, including feature extraction, selection and classification, to systematically assess a composite feature space by aligning EEG and musical features in time. The empirical results suggested that when EEG signals from the whole head were available, the inclusion of musical contents contributed little to the emotion classification model. On the contrary, if EEG dynamics only available from a small set of electrodes (likely the case in real-life BCI applications), the music modality tended to play a complementary role to enhance the EEG-based classification performance. To the best of our knowledge, no study has attempted to elucidate the roles of the EEG and music modalities in the emotion classification problem. The present study not only provided principles for building an EEG-based multimodal approach, but also revealed the fundamental insights into the interplay of the brain activity and musical contents in emotion modeling.

### Individual variability and commonality of the EEG dynamics for emotion classification

Individual variability has been reported in emotion regulations (Gross and John, 2003). Such variability may introduce the disparity of informative EEG patterns across individuals or subgroups (Lin et al., 2010a, 2011). To estimate the emotional states, it is plausible to expect a subject-specific classification model that well learned from an individual would have an optimal classification accuracy (Lin et al., 2010b). In the present study, the comparison in valence and arousal classification using subject-dependent and -independent features addressed this issue. The classification performance using the LFI-guided subject-independent EEG features (c.f. Figure 5) was notably worse than that using the subject-dependent EEG set (c.f. Figure 2). The commonality of the valence- and arousal-specific EEG features/electrodes from multiple subjects was rather small. There were only seven and four informative EEG features consistently appeared in over 15 of 26 subjects for valence and arousal classification, respectively (c.f. Table 3). These results suggested that the individual variability substantially affected emotion classification, especially for arousal scale, and thereby posed a great challenge to learning a subject-independent emotion model using only the EEG signals.

However, it is worth noting that exploring a consensus set of emotion-relevant EEG activity from multiple subjects is of great important to normative emotion research. In this study, the electrodes placed over the fronto-central region were relatively discriminative for most of subjects (c.f. Figure 6), which was in line with the previous studies (Altenmuller et al., 2002; Lin et al., 2010a). Over the brain region, the lateralized power asymmetry (in the left-right direction) well characterized the changes of emotional states, which may be supported by the role of the frontal cortical lateralization in emotion processing (Altenmuller et al., 2002; Allen et al., 2004). Specifically, the frontal theta asymmetry (FT7-FT8) and the fronto-central alpha asymmetry (FC3-FC4) associated with the valence scale was in line with other studies (Davidson, 1992; Aftanas et al., 2001; Schmidt and Trainor, 2001), whereas the fronto-central theta asymmetry (F7-F8 and FC3-FC4) related to the arousal scale was supported by Aftanas et al. (2004). Furthermore, several informative spectral asymmetries in the delta band for both emotional valence and arousal partially conformed to the previous works (Lin et al., 2010a,b). Accordingly, the index of rhythmic lateralization presumably better differentiated the brain activity into emotional states and acted consistently for multiple subjects, compared to the caudality (power asymmetry in the fronto-posterior direction) and individual spectra.

### The role of EEG and music modalities in emotion classification

The empirical results of this study evidently suggested that the inclusion of the acoustic characteristics of musical contents did not guarantee to complement EEG dynamics in the emotion classification problem. One key factor is that whether or not the EEG signals can be extracted from the whole brain and across entire frequency bands to encompass the full emotion-modulated spatio-spectral dynamics.

The optimized subject-dependent results showed that the EEG modality with and without the inclusion of the music modality were comparable in the performance (c.f. Figure 3) and tended to dominate the feature composition in the multimodality model (c.f. Figure 4). This indicated that the musical content brought very limited or redundant discriminative power to the classification of emotional responses. The aforementioned individual variability might explain such results. The music modality that lacks of correlates of internal psychophysiological reactions might more and less introduce conflicts with the brain signals, i.e., EEG modality, in reflecting the felt emotional responses. It is true that the listeners might not actually perceive and experience the same emotion as music tried to express (Gabrielsson, 2002). Accordingly, it is reasonable to conclude that if the informative EEG features can be obtained from the whole brain and entire frequency bands, the inclusion of musical contents barely contributed to the classification model. The multimodal approach might not be necessary.

However, in practical ABCI applications, an EEG cap with the whole-brain coverage might be impractical and unavailable in consumer-level headsets, e.g., the MindWave headset (NeuroSky, Inc.) and the Emotiv EPOC headset (Emotiv systems, Inc.). In this case, the EEG features measured by the electrodes sparsely placed at a certain brain region(s). The suboptimal EEG features returned very poor emotion classification performance (even lower than the random guessing, c.f. Figure 7). The music modality under this circumstance provided complementary information and replaced a set of EEG features with less discrimination power with the musical characteristics of timber and loudness (c.f. Figure 8). The musical dynamics tended to dominate the multimodal feature composition in the arousal scale as compared to valence. This phenomenon might be attributed to the fact that the music modality met a great challenge in modeling emotional valence (Macdorman et al., 2007; Yang et al., 2008b). This might also explain why the improvement in the classification performance was much noticeable in the arousal classification. Thus, the music modality was assumed to boost the EEG-based emotion classification performance if the EEG dynamics were substantially limited in certain brain regions.

### Informative musical characteristic for emotion classification

By manipulating musical structures, conveying emotions in music is intuitively plausible (Peretz et al., 1998; Schmithorst, 2005; Gomez and Danuser, 2007; Zatorre et al., 2007). Several neurophysiological studies that devoted to the brain correlates in musical perception and emotion perception reported that some music-modulated brain activity were known to intervene in emotion processing (Blood et al., 1999; Tsang et al., 2001; Khalfa et al., 2005). It is reasonable to expect that there is a considerable amount of EEG rhythmicity that is not only engaged in emotion processing but also modulated by music perception. Thus, the acoustic characteristics of musical contents and EEG dynamics could somehow perform complementarily. As shown in Table 4, previous neurophysiological and music signal processing studies supported our findings. Several neurophysiological studies found that mode and consonance were relevant to the distinction of emotion valence (Tsang et al., 2001; Sammler et al., 2007), whereas the harmonics processing was very closely associated with emotional affect and intensity (Schmithorst, 2005). From musical signal processing aspect, Yang et al. (2008b) reported that the valence scale was better characterized by the dissonance and pitch-related features, whereas the arousal scale was better modeled by timber features. This was in line to the findings of spectral dissonance and mode for valence scale and a timber element (8th MFCC) for arousal scale. Aljanaki et al. (2013) recently also documented that the most important feature in the distinction of the arousal scale was the loudness, which supported our findings in arousal scale. It is encouraging that the consistent findings of the musical structures were conducted with different musical datasets.

### Comparing the emotion classification results with previous works

Recent works that adopted the EEG-based multimodal approach are described here. Koelstra et al. (2012) proposed to use a decision-level fusion scheme to construct a multimodal pipeline (EEG, peripheral biosignals, music) for emotional valence, arousal and liking classification while watching music videos. The classification performance using the EEG signals were marginally worse than that using musical features for valence (EEG: 58%, biosignals: 63%, music: 62%, majority: 59%) and arousal (EEG: 62%, biosignals: 57%, music: 65%, majority: 64%) classification. The fusion of EEG and musical features resulted in an optimal classification accuracy around 63% marginally outperformed EEG modality only for arousal classification. In the same year, Soleymani et al. (2012a) also adopted the decision-level approach and explored an optimal fusion pair among EEG signals, peripheral biosignals and eye gaze for affective recognition during video appreciation. The classification performance using the EEG-Gaze fusion was better than single modality results for valence (biosignals: 46%, EEG: 57%, gaze: 69%, fusion: 76%, random: 34%) and arousal (biosignals: 46%, EEG: 52%, gaze: 64%, fusion: 68%, random: 36%) classification. The authors later performed a following-up study (Soleymani et al., 2012b) to compare the schemes for fusing EEG and gaze modalities at feature and decision levels. The authors reported that the decision-level fusion returned better classification results compared to single modalities for valence (EEG: 50%, eye: 67%, decision: 69%, random: 33%) and arousal (EEG: 62%, eye: 71%, decision: 76%, random: 33%) classification, where the feature-level fusion (valence: 58%, arousal: 66%) just outperformed the EEG modality. A year later, Koelstra and Patras (2013) similarly assessed the feasibility of using the feature- and decision-based multimodality (EEG dynamics and facial expression characteristics). The authors documented that the feature-level fusion in general marginally improved the performance against single modalities for valence (EEG: 72%, face: 65%, fusion: 73%, majority: 62%) and arousal (EEG: 68%, face: 68%, fusion: 69%, majority: 62%) classification, whereas the fusion-level approach using an optimal weighting scheme led to more convincing improvement (valence: 74%, arousal: 72%). In the present study, the feature-level multimodal approach (EEG and musical features) was adopted to validate its feasibility of emotion classification in music listening. The empirical result showed that the subject-dependent multimodal approach marginally outperformed the single modalities for valence (EEG: 76%, music: 65%, fusion: 77%, majority: 63%) and arousal (EEG: 74%, music: 66%, fusion: 76%, majority: 61%) classification, whereas the subject-independent multimodal approach provided more convincing improvement for valence (EEG: 61%, fusion: 67%) and arousal (EEG: 58%, fusion: 67%) classifications.

It is worth mentioning that the comparison only based on classification accuracy might not be fair as a variety of factors might affect the classification results, such as but not limited to experimental conditions, stimulus types, multimodal sources, and signal processing steps. Thus, for a fair comparison in multimodality, this study summarized the differences between this and another related work (Koelstra et al., 2012) that also focused on the multimodality of EEG and musical dynamics. The obtained subject-dependent results of this study were evidently higher than theirs by at least 10%, whereas the subject-independent results of this study were marginally higher by around 3%, yet with fewer EEG features and electrodes. Despite the disparity in the selected musical features, the music modality results for emotional valence and arousal were comparable. Furthermore, compared to the studies using solely EEG modality (Koelstra et al., 2012; Soleymani et al., 2012a,b; Koelstra and Patras, 2013), the proposed subject-dependent EEG features (MESH) should be comparable to or even better than previous reports. Instead, the classification performance using the proposed subject-independent EEG set might only compare favorably to the study (Koelstra et al., 2012).

### Outperformed EEG patterns for emotion classification

The MESH features in conjunction with the F-score feature selection produced a compact set of informative features and consequently optimized the classification performance, compared to others (DLAT, DCAU, and PSD) (c.f. Figure 2). The performance improvement might be attributed to the fact that emotion processing might accompany the EEG dynamics that varied distinctly within and between brain regions (Schmidt and Trainor, 2001; Aftanas et al., 2004; Sarlo et al., 2005; Sammler et al., 2007; Lin et al., 2010b). The MESH features that composed of two-directional power asymmetry (laterality and caudality) and individual spectra over the scalp allow seeking an optimal set for constructing a classification model for each individual. As referring to its feature composition (c.f. Figure 4), both DLAT and DCAU apparently dominated the EEG composition against the PSD. Specifically, the DLAT consistently appeared in multiple subjects (c.f. Table 3). These results suggested that the features depicting the directional spectral differences between brain regions might be of importance in the EEG-based emotion modeling.

### The choice of EEG electrode reference

The EEG signals analyzed in this study were recorded with the reference to the linked mastoids. The recorded potentials over the mastoids were conventionally believed to be neutral to the measured neural activities of interest, which were also adopted in previous music studies (Koelsch et al., 2007; Sammler et al., 2007). However, few reports demonstrated that the linked mastoids reference might introduce non-neutrality to the recorded EEG signals and distort the EEG spectra (Yao, 2001; Marzetti et al., 2007; Qin et al., 2010). Comparing the effects of different reference strategies on emotion classification is an important issue, but it is beyond the scope of this study. Interested readers can refer to the studies on reference techniques by Yao (2001), Marzetti et al. (2007), Qin et al. (2010).

### Future direction

Future efforts can be devoted to augment the multimodal classification performance as follows. First, data-driven approach, e.g., principal component analysis (Lin et al., 2009) and independent component analysis (Lin et al., 2010a), might be feasible to further elaborate the EEG spatio-spectral dynamics associated with implicit emotional responses. Second, advanced music signal processing techniques can be incorporated to extract other musical characteristics, e.g., rhythm. Lastly, the decision-level multimodal fusion has been reported to obtain convincing classification performance improvements over the feature-level fusion (Soleymani et al., 2012b; Koelstra and Patras, 2013). Following the explored EEG and musical features of this study, the fusion at the decision level can be further explored and compared.

### Conflict of interest statement

The authors declare that the research was conducted in the absence of any commercial or financial relationships that could be construed as a potential conflict of interest.
